# Use of an Electrochemical Split Cell Technique to Evaluate the Influence of *Shewanella oneidensis* Activities on Corrosion of Carbon Steel

**DOI:** 10.1371/journal.pone.0147899

**Published:** 2016-01-29

**Authors:** Robert Bertram Miller, Anwar Sadek, Alvaro Rodriguez, Mariano Iannuzzi, Carla Giai, John M. Senko, Chelsea N. Monty

**Affiliations:** 1 Department of Biology, The University of Akron, Akron, OH 44325, United States of America; 2 Integrated Bioscience Program, The University of Akron, Akron, OH 44325, United States of America; 3 Department of Chemical and Biomolecular Engineering, The University of Akron, Akron, OH 44325, United States of America; 4 Department of Geosciences, The University of Akron, Akron, OH 44325, United States of America; University Hospital of the Albert-Ludwigs-University Freiburg, GERMANY

## Abstract

Microbially induced corrosion (MIC) is a complex problem that affects various industries. Several techniques have been developed to monitor corrosion and elucidate corrosion mechanisms, including microbiological processes that induce metal deterioration. We used zero resistance ammetry (ZRA) in a split chamber configuration to evaluate the effects of the facultatively anaerobic Fe(III) reducing bacterium *Shewanella oneidensis* MR-1 on the corrosion of UNS G10180 carbon steel. We show that activities of *S*. *oneidensis* inhibit corrosion of steel with which that organism has direct contact. However, when a carbon steel coupon in contact with *S*. *oneidensis* was electrically connected to a second coupon that was free of biofilm (in separate chambers of the split chamber assembly), ZRA-based measurements indicated that current moved from the *S*. *oneidensis*-containing chamber to the cell-free chamber. This electron transfer enhanced the O_2_ reduction reaction on the coupon deployed in the cell free chamber, and consequently, enhanced oxidation and corrosion of that electrode. Our results illustrate a novel mechanism for MIC in cases where metal surfaces are heterogeneously covered by biofilms.

## Introduction

Microbiologically influenced corrosion (MIC) is one of the most insidious forms of corrosion and results in nearly 50% of all corrosion costs, which can add up to as much as 140 billion USD in the US alone [1, 2]. In general, MIC is controlled by reactions occurring within the metal substrate or at the substrate/electrolyte interface in the response to microbial metabolism. These metabolic activities can include but are not limited to, direct metabolism of metals/alloys, metabolism of corrosion-products on the metal/alloy, or secretion of ligands or other metabolites that enhance metal dissolution and/or metal oxidation [3–15]. Of particular importance in MIC is the development of biofilms [10–15]. Microbial activities in biofilms may give rise to chemical conditions (e.g. pH, O_2_ or metabolite concentrations) that are dramatically different from those of the bulk fluid. These activities exert considerable control on the chemistry of fluids immediately adjacent to the metal surface [10, 14, 16, 17]. Microbial activities can also limit the contact between O_2_ or other corrosive compounds and the metal surface, inhibiting corrosion processes [18–24]; however, heterogeneous biofilms may induce corrosion through formation of differential aeration and concentration cells on the surface of the metal [25–29]. Given the diversity of mechanisms by which microbial activities may enhance and/or inhibit corrosion, it is necessary to develop a clear mechanistic understanding of the factors involved in MIC. Ultimately, this information may be used to develop preventive methods and monitoring tools for MIC.

In this work, we used *Shewanella oneidensis* MR-1 as an organism to model MIC processes principally due to its metabolic versatility, including aerobic respiration and dissimilatory Fe(III) reduction [18, 30, 31]. Additionally, while the influences of *Shewanella* species (as model Fe(III) reducing bacteria) on carbon steel corrosion have been examined, evidence for these influences remains equivocal [18, 27, 31]. On one hand, it has been suggested that *Shewanella* may inhibit steel corrosion by reductively dissolving Fe(III) (hydr)oxide protective layers, with the resultant dissolved Fe(II) scavenging O_2_, and limiting interaction between O_2_ and the metal surface [18, 27]. On the other hand, Fe(III) respiration may serve to reductively dissolve the Fe(III) (hydr)oxide protective layer, thus enhancing deterioration of the steel [27].

In order to electrochemically monitor corrosion of carbon steel, we used zero-resistance amperometry (ZRA) in a split-cell (referred to as “split-chamber” hereafter) technique to evaluate the roles, potential mechanisms, and electrochemical signatures associated with MIC in the presence/absence of *Shewanella*. Variations of this approach have been widely used to interrogate mechanisms of corrosion, and have been proposed as monitoring tools for MIC [32–37]. The ZRA split-chamber technique allows the measurement of current flow and coupling potential (E_coupl_) between two identical or dissimilar materials [32–36, 38, 39]. In addition, the environmental conditions of each chamber can be manipulated independently. For example, by including bacteria in one chamber and sterile medium in the other chamber of the split-chamber assembly we are able to mimic the conditions of heterogeneous metal surface coverage, which are believed to lead to localized corrosion [30, 31, 40, 41, 42]. Thus, the split-chamber setup can provide mechanistic information that is impossible to obtain using conventional electrochemical techniques (e.g. linear polarization, electrochemical impedance spectroscopy) [39]. As such, the ZRA based measurements may provide mechanistic insights into the role of dissimilatory Fe(III) reducing bacteria in corrosion of carbon steel.

ZRA-based current and potential measurements were correlated with other measurements, such as changes in aqueous chemistry or metal properties, to elucidate corrosion and corrosion inhibition mechanisms [25, 42]. Estimations of corrosion based on electrochemical measurements were confirmed by weight loss analysis. As opposed to other split chamber-based approaches to evaluate MIC [37, 39], in the experiments described here, no effort was made to exclude O_2_ from either of the WE1 or WE2 chambers, in order to monitor aerobic reactions. The only differences between the systems were the presence or absence of *S*. *oneidensis*, and a microbiologically-induced redox gradient was allowed to develop. *S*. *oneidensis* ability to transfer electrons was monitored under redox conditions [43–45]. As such, this work may provide a mechanism for, and approach to monitoring corrosion of metals that span redox regimes, geochemical conditions (e.g. pipes) or experience conditions of alternating O_2_ availability (e.g. coastal structures).

## Materials and Methods

### Bacterial cultivation

*Shewanella oneidensis* MR-1 was routinely grown on a solid or liquid tryptic soy media (TSA and TSB, respectively) consisting of tryptic soy powder (20 g/L) and bacto agar (15 g/L for solid medium). Experiments were conducted in a minimal medium used by Myers and Nealson [31] that included: 50 mM 4-(2-hydroxyethyl)-1-piperazineethanesulfonicacid (HEPES), 9.0 mM (NH_4_)_2_SO_4_, 5.7 mM K_2_HPO_4_, 3.3 mM KH_2_PO_4_, 2.0 mM NaHCO_3_, 1.0 mM MgSO_4_, 0.49 mM CaCl_2_, 0.05 g/L yeast extract, vitamins and trace metals [46], 15 mM sodium lactate, casamino acids (0.1 g/L), L-arginine HCl (20 mg/L), and L-glutamate (20 mg/L). In some cases, lactate was omitted from the medium. Cells were grown for approximately 24 hours in TSB to late log phase at room temperature and shaking at 120 rpm. Cells were harvested by centrifugation, washed with lactate-free minimal medium (above), and resuspended in the same medium. *S*. *oneidensis* growth was determined based on optical density measurements at 600 nm in a Helios UV/VIS spectrophotometer. Biofilm development was observed in batch corrosion incubations (described below) using confocal microscopy (Olympus, FV1000 Confocal laser scanning microscope). Cells were stained using Life Technologies (ThermoFisher, Waltham, MA) Bac-Light bacterial viability and counting kit reagents according to the manufacturer’s instructions [47].

### Corrosion incubations

Carbon steel (UNS G10180) samples were ground using progressively finer SiC papers including 240, 320, 400, and 600 grit, as described in ASTM standard E1558 [48]. Samples were sterilized by placing them in a glass chamber filled with non-reactive nitrogen. The chamber was then placed in an oven at 160°C for 4 hours. This process sterilizes the metal while minimizing alteration of the metal surface, which occurs during other standard sterilization approaches (e.g. autoclaving) [49]. Briefly, flat steel coupons (for batch incubations) were placed in serum tubes that were sealed with thick butyl rubber stoppers with aluminum crimp seals, while cylindrical coupons (for ZRA measurements) were placed in the split chamber assembly (described below). Air was evacuated from serum tubes or split chamber assemblies, and replaced with N_2_. Evacuation and N_2_ replacement were conducted three times, after which, the serum tubes or cell assembly was placed in an oven at 160°C for four hours.

Batch corrosion experiments were conducted in 125 mL flasks containing 50 mL of minimal medium, and sterilized coupons were added to the medium aseptically. A 10% volume inoculum of TSB-grown *S*. *oneidensis* was added to minimal medium at an optical density of 0.912 at 600 nm, after being washed in minimal media three times. Cultures were incubated at 22°C and with shaking (120 rpm). Samples were periodically recovered and growth was determined based on the optical density of the cultures, which was determined as described above. Cells were removed from suspension by centrifugation, and the supernatant was removed for subsequent measurements of dissolved Fe(II) and lactate concentrations (described below). Samples intended for measurement of dissolved Fe(II) were preserved in 0.5 M HCl.

To evaluate steel corrosion in the split chamber format, two glass cells were assembled with 250 mL of minimal medium in both chambers, which were separated by a salt bridge consisting of a cation exchange membrane (CMI-7000S; Membranes International Inc.; Ringwood, NJ) that was primed in sterile 5% NaCl solution at 40°C for 24 hours prior to use. Primed membranes were aseptically inserted into split chamber assemblies after sterilization of the assemblies (described above). Polished working electrodes (referred to as WE1 and WE2) were included in the two cells, with a saturated calomel electrode (SCE) reference electrode deployed in the cell containing WE1. Each WE had an exposed area of 0.5 cm^2^. Current and potential were recorded using a Gamry Reference 600 potentiostat/galvanostat in zero resistance ammeter (ZRA) mode. In this configuration, a positive current represents flow of electrons from WE1 to WE2. To confirm the sign convention for current, experiments were conducted with Al (WE1) and Cu (WE2) coupons in an identical configuration (S1 Fig). The resulting galvanic current from this experiment was positive, verifying our sign convention based on electrochemical thermodynamics for this system (S2 Fig). Galvanic potential readings were collected at in two minute intervals during operation of the split-chamber experiments. For experiments that included cells, chamber WE1 received an inoculum of concentrated *S*. *oneidensis* cells that were prepared as described above. Where appropriate, lactate was re-amended to the WE1 chamber from a sterile 150 mM stock solution to achieve a concentration of approximately 8 mM. Samples were periodically removed from both cells of the assembly as described above to measure cell density, pH, dissolved Fe(II), and lactate. At the conclusions of batch and ZRA experiments, the steel coupons were removed subjected to weight loss analysis (described below).

Corrosion rates were determined by weight loss analysis (WLA) using ASTM method G01-03 [50]. Samples were rinsed in deionized water, wire brushed, and, then, immersed in Clarke’ Reagent (1000 mL 12.1 M HCl, 20 g antimony trioxide, and 50 g stannous chloride) for 30 seconds to remove surface oxides. After the Clarke’s Reagent bath, the coupons were rinsed with DI water, dried, and weighed. The Clarkes’ reagent wash, DI wash, and weighing were repeated until no mass was lost between wash cycles, indicating that all oxides were removed [50]. The total mass loss was recorded indicating the physical weight that was lost due to corrosivity of the environment. Corrosion rate was calculated using Eq 1

(CR= (W*K)/(D*A*t)) (1)

where *CR* represents the corrosion rate in mm/yr, *K* (8.76 × 10^4^) is a dimensionless constant, *W* is the mass loss in grams, *A* is the exposed surface area in cm^2^, *T* is exposure time in hours and *D* is the density of carbon steel UNS G10180 in g/cm^3^ [50].

### Analytical techniques

Dissolved Fe(II) was quantified by ferrozine assay [51], and lactate was quantified by high-performance liquid chromatography, using and a Shimadzu LC-10A HPLC system (Shimadzu Scientific Instruments, Inc.; Columbia, MD) equipped with an Aminex HPX-87H column (300 mm × 7.8 mm; Bio-Rad Laboratories, Inc.; Hercules, CA) with UV (254 nm) detection (SPD-10A). A mobile phase of 0.008 N H_2_SO_4_ was used at a flow rate of 0.6 mL/min.

## Results

### Batch experiments

**Fig 1:**
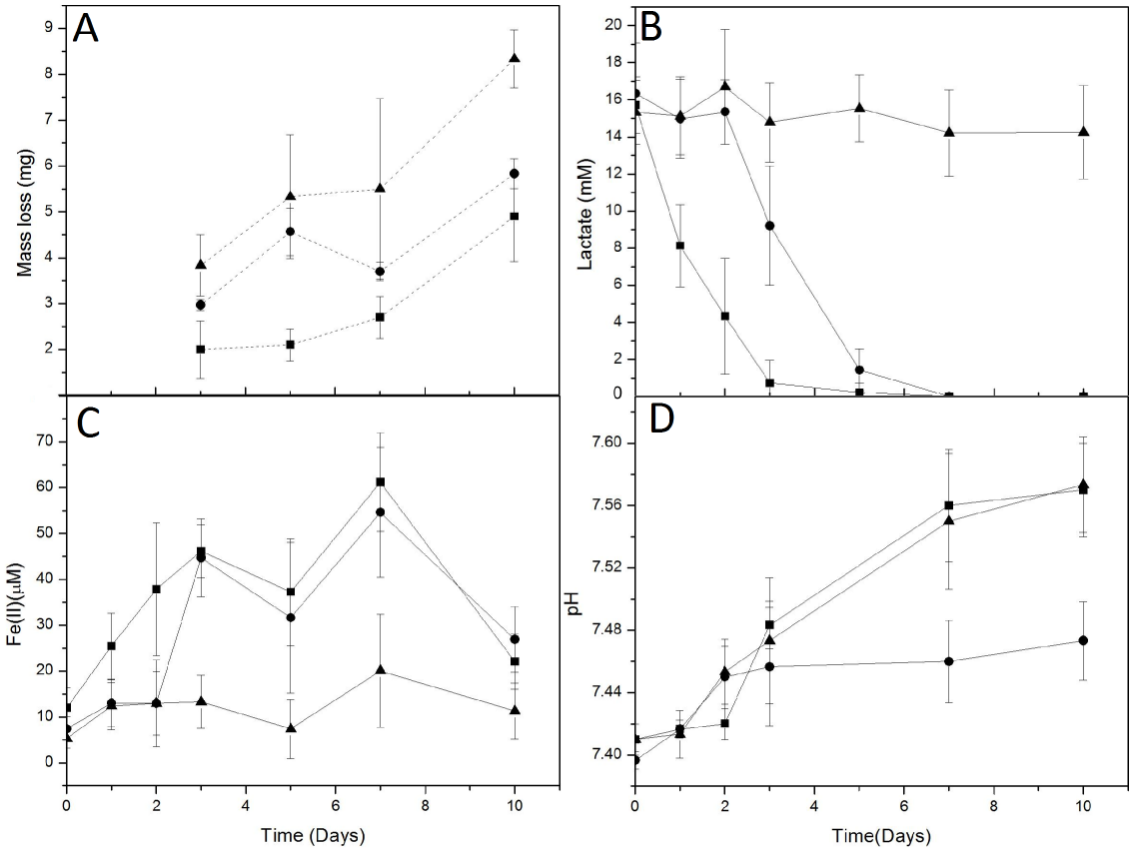
Mass loss from carbon steel coupons (A), lactate concentration (B), dissolved Fe(II) concentration (C), and pH (D) in incubations that contained growth medium and steel coupons, and were (■) inoculated with *S*. *oneidensis* immediately, (●) inoculated with *S*. *oneidensis* after 2 day preincubation of the coupon, or (▲) in uninoculated medium. Error bars represent one standard deviation of triplicate incubations.

Batch experiments were conducted to establish a baseline for corrosion rate in the absence and presence of *S*. *oneidensis*. In experiments that included carbon steel coupons incubated under static conditions, more extensive corrosion was observed in uninoculated medium than in incubations that included *S*. *oneidensis* (Fig 1). In batch experiments, activity of *S*. *oneidensis* was indicated by lactate depletion and accumulation of dissolved Fe(II) (Fig 1). pH increase was indicative of reduction of Fe(III) (hydr)oxide phases (Fig 1). Addition of *S*. *oneidensis* to incubations that did not initially receive inoculation arrested further corrosion (Fig 1). These results indicate that the activities of *S*. *oneidensis* in direct contact with carbon steel limit corrosion of the carbon steel.

### Split-chamber incubations

A series of different environments along with biotic and abiotic controls were then tested to determine the effect of bacteria on the corrosion of carbon steel coupons during ZRA testing (Table 1). Corrosion rates of each coupon were reported as well as the ratio of corrosion rates between WE2 and WE1 (Table 1). Similar rates and extents of corrosion were observed for WE1 and WE2 during ZRA experiments that did not include cells in either chamber (Table 1) and the corrosion rate ratio (CR_WE2_/CR_WE1_) was approximately one, indicating the same redox reactions (i.e. Fe(0) oxidation coupled to O_2_ reduction, Reactions 1 and 2), were occurring in both chambers. Likewise, when both chambers received inoculation with *S*. *oneidensis*, minimal current was observed (Table 1), indicating little electron transfer between the two chambers. Taken together, these results indicate that when both compartments were under identical environmental conditions, there was not enough electrochemical driving force to measure a net current between working electrodes. This scenario resembled the conditions shown in electrochemical noise analysis (ENA) [52, 53].

**Table 1:** Summary of split-chamber ZRA experimental conditions and electrochemical and corrosion characteristics during the incubations.

Environment		Carbon Source	Inoculation	Inoculation Time (hr)	CR (mm/yr)	CRR^a^	Imax (μA)	ECoupl (mV_SCE_)
1	WE1	Lactate	*S*. *oneidensis*	48	0.07	2.4	16	-720
	WE2	Lactate	*Sterile*	N/A	0.17			
	WE1	Lactate	*S*. *oneidensis*	48	0.08	2.23	12	-712
	WE2	Lactate	Sterile	N/A	0.19			
2	WE1	Lactate	*S*. *oneidensis*	0	0.04	2.25	6.15	-720
	WE2	Lactate	Sterile	N/A	0.1			
3	WE1	None	*S*. *oneidensis*	48	0.09	2	0.15	-708
	WE2	None	Sterile	N/A	0.18			
4	WE1	None	*S*. *oneidensis*	0	0.07	1.3	0.91	-727
	WE2	None	Sterile	N/A	0.09			
5	WE1	Lactate (+)	*S*. *oneidensis*	48	0.04	6.36	31	-690
	WE2	Lactate	Sterile	N/A	0.23			
C1	WE1	Lactate	*S*. *oneidensis*	48	0.09	1.11	0.88	-730
	WE2	Lactate	*S*. *oneidensis*	48	0.1			
C2	WE1	Lactate	*S*. *oneidensis*	0	0.02	-	1.21	-718
	WE2	Lactate	*S*. *oneidensis*	0	0.06			
C3	WE1	Lactate	Sterile	N/A	0.1	1.11	0.87	-690
	WE2	Lactate	Sterile	N/A	0.11			
	WE1	Lactate	Sterile	N/A	0.08	1.1	0.97	-686
	WE2	Lactate	Sterile	N/A	0.09			
C4	WE1	None	Sterile	N/A	0.19	1.08	0.32	-740
	WE2	None	Sterile	N/A	0.21			

^a^CRR is corrosion rate ratio, calculated as CR_WE2_/CR_WE1._

When WE1 was inoculated with bacteria in the presence of lactate, however, the corrosion rate ratio (CR_WE2_/CR_WE1_) was > 2.25, with a maximum coupled current approximately 10 times higher than control experiments. It should be noted that couple potentials were similar to sterile controls. However, when WE1 was inoculated with bacteria in the absence of lactate, the corrosion rate ratio (CR_WE2_/CR_WE1_) was between 1.4 and 2 (on par with the ZRA controls), with minimum couple currents. In batch experiments the ratio of corrosion rate of uninoculated samples compared to inoculated samples was approximately 1.5, indicating that connecting the metal samples through a ZRA in a split-chamber format in the presence of lactate caused an increase in corrosion on the biofilm-free WE2. Additionally, lactate was not consumed and Fe(II) was not produced in environments without bacteria (S1 Table). The role of lactate as a corrosion inhibitor was indicated by sterile controls supplied with lactate having half the corrosion rate as non-lactate supplied controls (Table 1). The inhibitory properties of lactate have been previously reported [54].

**Fig 2:**
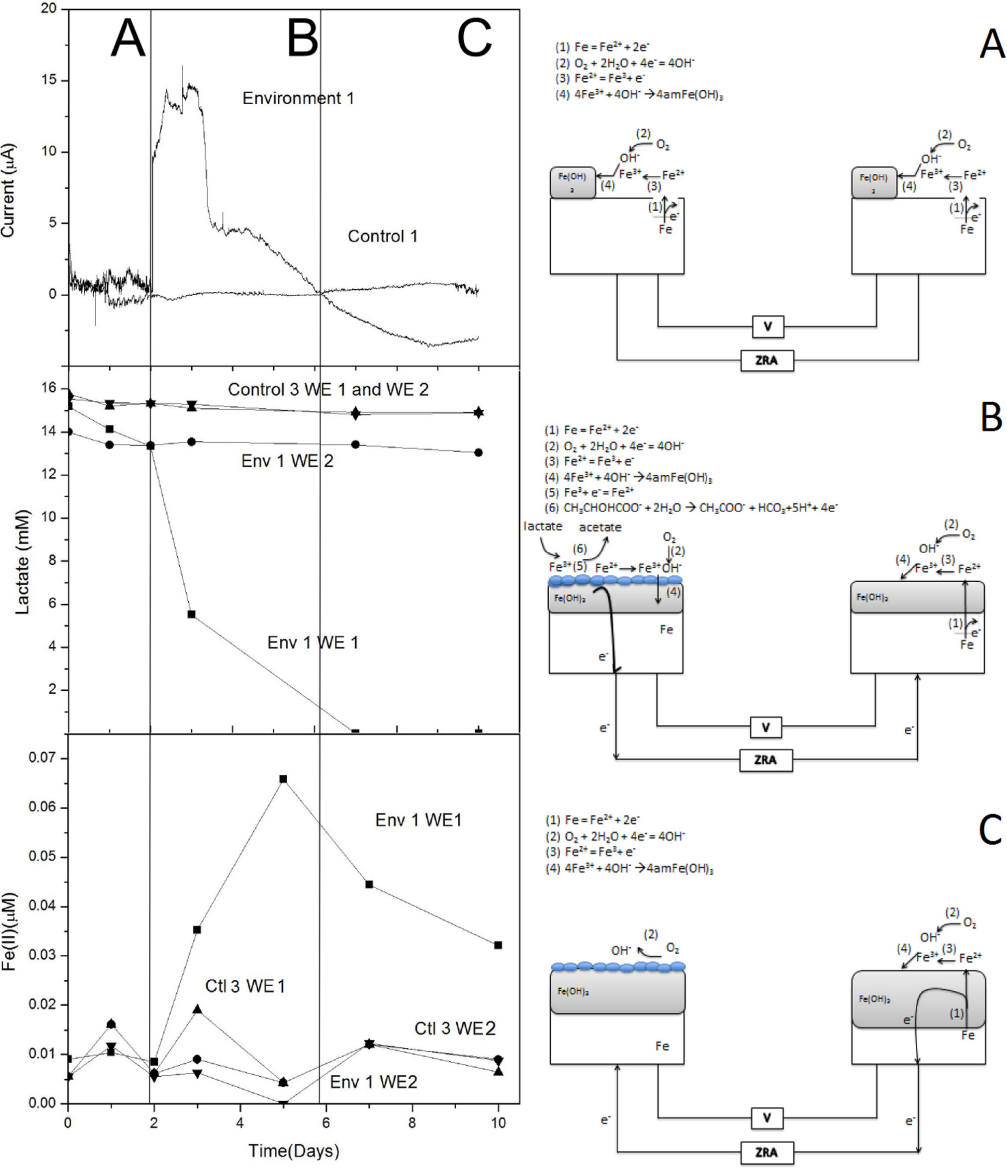
Galvanic current, lactate concentrations, and Fe(II) concentrations measured in split-chamber incubations. Env. 1 indicates incubations that received *S*. *oneidensis* in the WE1 chamber after 2 d of preincubation, and Control 3 indicates incubations that did not receive inoculation. Lactate and dissolved Fe(II) concentrations are shown in both the WE1 and WE2 chambers. Panels A-C illustrate configuration of split-chamber incubations and ZRA measurements, as well the proposed redox processes that are hypothesized to occur during different phases (A-C) of the incubations.

To better interpret these results, we evaluated the current over time during ZRA experiments. In ZRA split-chamber tests, before inoculation, WE1 and WE2 were exposed to identical conditions and behaved similarly to control experiments (Fig 2). During this period, Fe(0) oxidation (Reaction 1) and O_2_ reduction (Reaction 2) reactions occurred at the surface of both electrodes. At near neutral pH, the Fe(II) formed via Reaction 1 was further oxidized via Reaction 3. Coupled with the O_2_ reduction reaction, the reactions formed an amorphous iron hydroxide layer with a mixed Fe(II)/Fe(III) oxidation state [54]. During this period (A), little net electron exchange between chambers was observed, as evidenced by the limited galvanic current (Fig 2).

$$Fe=\;Fe^{2+}+2e^{-},E=0.44\;V-0.0295{\text{log}}_{10}\left[Fe^{2+}\right]$$ Reaction 1

$$O_{2}+2H_{2}O+4e^{-}=4OH^{-},\;E=1.23-0.059pOH$$ Reaction 2

$$Fe^{2+}=Fe^{3+}+e^{-},\;E=\;-0.77V-0.059{\text{log}}_{10}\left[Fe^{3+}/Fe^{2+}\right]$$ Reaction 3

Upon inoculation of WE1, an increase in positive galvanic current was observed concurrent with lactate depletion and dissolved Fe(II) accumulation (Env 1, WE1; Fig 2). Little change in lactate and dissolved Fe(II) concentration was observed in the uninoculated chamber (WE2). Comparing these results to the increased corrosion rate suggests that the Fe^2+^ generated may be part of an amorphous mixed valence (Fe^2+^/Fe^3+^) protective layer. During this period (B), *S*. *oneidensis* reduced the Fe(III) formed on the surface of WE1 (Reaction 4). The Fe(II) formed during lactate metabolism was then oxidized to reform iron hydroxide, with the excess Fe(II) diffusing into the bulk solution (Fig 2). Coupling the electrochemical oxidation of Fe(II) with biological reduction of Fe(III) and O_2_ created a sustainable iron oxidation/reduction cycle. O_2_ was depleted by both enzymatic activity and by reaction with Fe(II) at the surface of WE1, producing a differential aeration scenario in which excess electrons produced during lactate oxidation travel through the ZRA from WE1 to WE2 (Fig 2). The electroneutrality of WE1 is therefore maintained by the biological cycle, where lactate oxidation can proceed at a faster rate due to the increase the O_2_ reduction reaction on WE2. As the O_2_ reduction reaction increases, an increase in Fe(0) oxidation on WE2 occurs in order to maintain local electroneutrality in the WE2 chamber, leading to greater corrosion of WE2 compared to WE1 (as determined by weight loss; Table 1). Additionally, lactate consumption far exceeded that necessary to produce the 60 μM Fe(II) observed, further indicating that lactate oxidation by *S*. *oneidensis* was coupled to both O_2_ and Fe(III) reduction in the WE1 chamber. This activity has been previously suggested to limit corrosion of steel, whereby microbiological O_2_ respiration limits Fe(0) oxidation (Fig 2B), while biogenic Fe(II) (from bioreduction of Fe(III) (hydroxide protective layer) further limits interaction between O_2_ and Fe(0) [18, 28].

$$CH_{3}CHOHCO^{-}+2H_{2}O=CH_{3}COO^{-}+HCO_{3}+5H^{+}+4e^{-}$$ Reaction 4

Upon complete depletion of lactate, galvanic current became negative (Fig 2C) indicating the flow of electrons was from WE2 to WE1. During this period (C), the depletion of lactate shut down the Fe oxidation/reduction cycle maintained by *S*. *oneidensis* (Reaction 4, Fig 2), allowing O_2_ to return to the surface and participate in surface reactions. The bacterial biofilm acted as a protective layer, inhibiting further Fe(0) oxidation causing electrons from WE2 to be drawn towards WE1 to participate in the O_2_ reduction reaction, cathodically protecting WE1 and leading to further corrosion of WE2.

**Fig 3:**
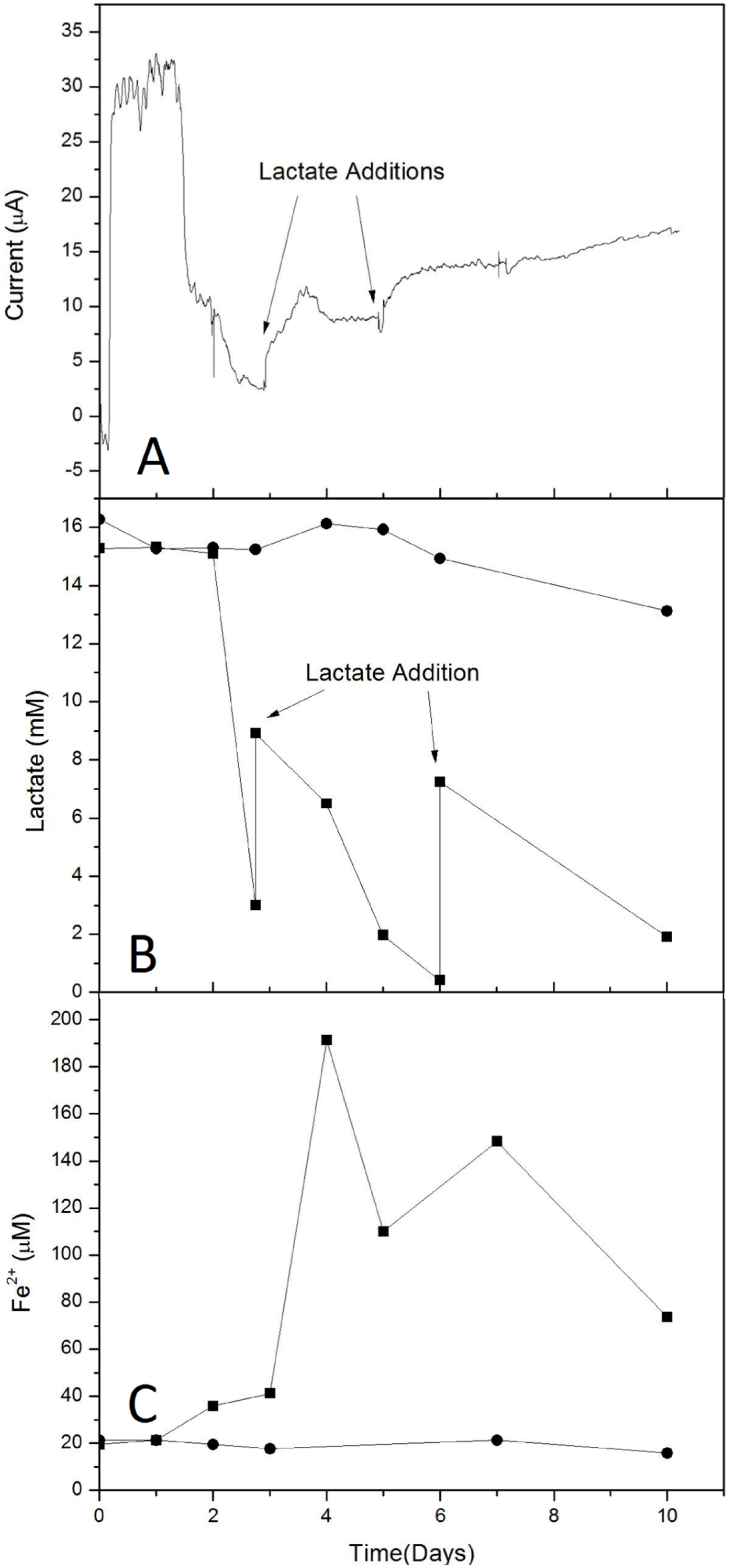
Galvanic current, lactate concentrations, and Fe(II) concentrations (panels A-C, respectively) measured in split-chamber incubations. Lactate and Fe(II) concentrations in WE1 (which received *S*. *oneidensis* and lactate amendments) and WE2 chambers are depicted with ■ and ●, respectively.

To test the hypothesis that lactate metabolism in the WE1 chamber created a protective barrier against O_2_ interactions with the steel surface of WE1, similar experiments were carried out in which *S*. *oneidensis* was added to the WE1 chamber, but lactate was periodically replenished in that chamber. Upon addition of *S*. *oneidensis* to the WE1 chamber, an increase in current was observed (Fig 3A) in a fashion similar to that observed previously (Fig 2). The increase in current was concurrent with lactate consumption and Fe(II) formation (Fig 3B and 3C). As lactate was depleted, current again decreased, but upon amendment with additional lactate, current again increased (Fig 3A). However, the increase in current was not of the same magnitude as that observed with the initial addition of cells. The addition of lactate to the WE1 chamber led to greater Fe(III) reduction than was observed during the initial period of lactate consumption, and the Fe(II) concentration remained high after the second addition of lactate, which again led to an increase in current (Fig 3A–3C). Replenishment of lactate enhanced microbial metabolism on the WE1 side, enhanced current, and led to more corrosion of WE2, and slightly less corrosion of WE1 resulting in a corrosion rate ratio of 6.4 (Table 1). These findings support the hypothesis that microbial lactate oxidation in one chamber provided a driving force for increased O_2_ reduction reaction in WE2, resulting in enhanced corrosion of WE2 and inhibited corrosion of WE1 as explained above.

**Fig 4:**
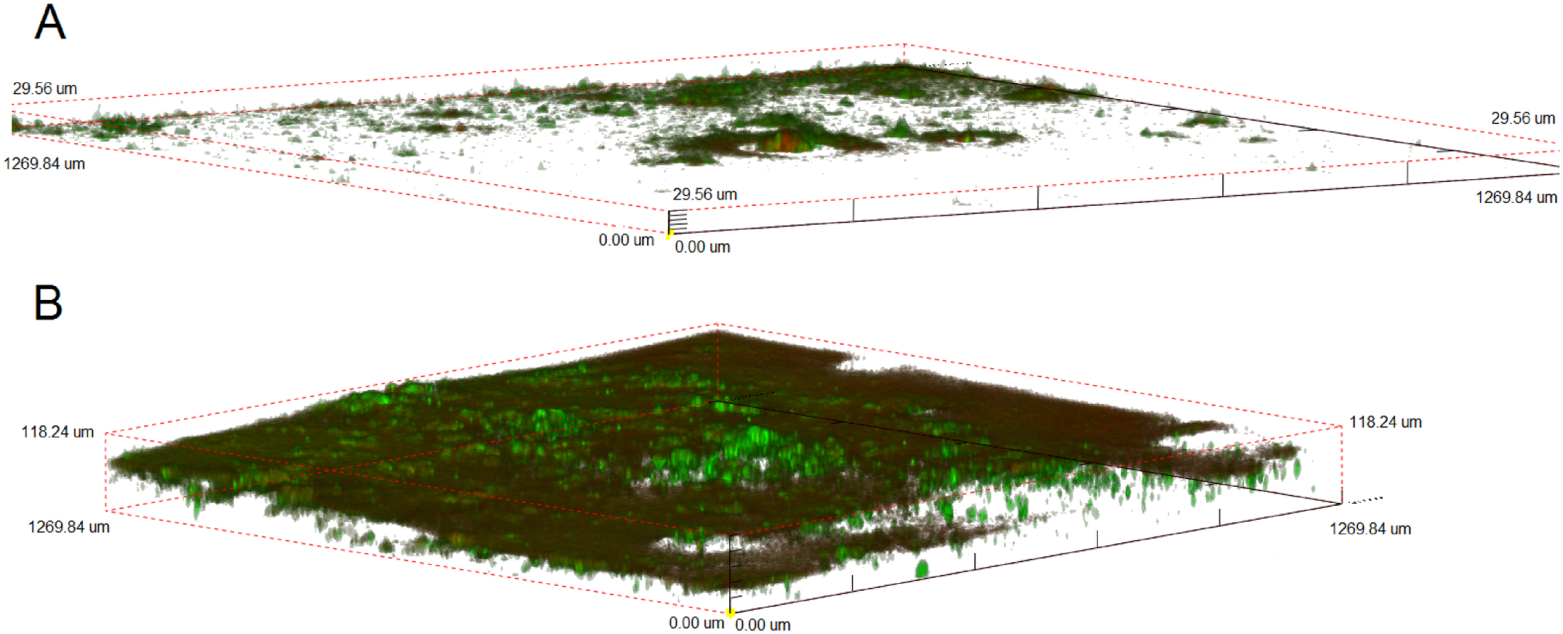
Confocal laser scanning micrographs of Bac-Light-stained *S*. *oneidensis* incubated for 72 h on carbon steel coupons in medium without (A) and with (B) lactate.

Based on the corrosion rate ratio numbers presented in Table 1, it is clear that the presence of bacteria is necessary for corrosion enhancement of the uncovered WE2 compared to controls. This corrosion enhancement is further accelerated in the presence of lactate. To ensure that WE1 was covered by a uniform biofilm, confocal images indicated the presence of a robust biofilm on steel coupons in the presence of lactate, while only minimal coverage of coupons was observed in the absence of lactate (Fig 4), similar to previous results [55, 56]. Lactate [and Fe(III)] metabolism, along with the magnitude and direction of current (from WE1 to WE2) suggest that activities of *S*. *oneidensis* biofilms lead to the development of excess reducing power on the WE1 side of the split-chamber assembly, which migrated to, and reacted on WE2, leading to greater oxidation of Fe(0) by O_2_ reduction (Fig 1B). Subsequently, corrosion rate ratios (WE2/WE1) of >2.25 were consistently observed in the environments containing both bacteria and lactate (Table 1), and similar increases in current were observed upon inoculation of the WE1 chamber (maximum current, Table 1). In the absence of lactate, the bacteria provided some corrosion protection via biofilm formation, indicating that the bacteria alone may reduce Fe(III) on precorroded metal surfaces. This is further indicated by the low Fe(II) concentration in solution (S1 Table). The corrosion protection in the presence of bacteria without lactate was comparable to batch experiments not connected through a ZRA (Fig 1).

## Discussion

Previous work to evaluate the influences of Fe(III) reducing bacteria on corrosion of carbon steel are inconclusive. While some authors suggested that the activities of *Shewanella* species may inhibit corrosion [16, 43, 57, 58] others have shown that *Shewanella* species enhanced corrosion of steel [31, 32]. In the current work, we have observed that, depending on the presence and distribution of biofilm, the activities of *S*. *oneidensis* both inhibit and enhance corrosion of steel. When *S*. *oneidensis* has direct contact with steel, corrosion is inhibited, likely due to O_2_ scavenging by a combination of metabolic consumption and by reaction with biogenic Fe(II) [16, 31, 32, 57]; however, when the steel surface in contact with *S*. *oneidensis* is electrically connected to an uncovered portion of steel, the corrosion of the uncovered portion is enhanced by, at least, a factor of 2. Such a scenario could be quite common in “real world” situations, where biofilm coverage is likely to be heterogeneous [6]. This heterogeneous surface coverage by biofilms and consequent development of regions of localized corrosion on the metal surface has been suggested as the cause for localized corrosion [13, 26, 32, 37, 59], and in the experiments reported here, we have been able to illustrate this phenomenon experimentally and provide a possible mechanism.

We have also shown that the activities of *S*. *oneidensis* induce conditions that enhance corrosion of uncovered metal surfaces. Given that lactate oxidation was supported not only by biological activities but also by the O_2_ reduction reaction on WE2, iron dissolution in WE2 occurred at a faster rate than in the uncoupled scenario so that local charge neutrality was maintained in the WE2 chamber. To further verify this mechanism, little current was observed when no lactate was present. In the absence of lactate, there was less driving force for reduction of the Fe(III) hydroxide layer (“Environment 4” in Table 1). However, the nature of this association is not completely clear, since correspondingly greater current was not observed with the high Fe(II) accumulation that resulted from lactate reamendment (Fig 3).

### Implications

This work experimentally illustrates a mechanism for the localized corrosion of uncovered metals at the biofilm/metal interface and it is different from what has been suggested previously. Similar ZRA based approaches have been exploited to elucidate mechanisms of MIC and proposed as MIC detection and monitoring approaches [34, 35, 37, 60]. It is notable that in many of these cases, a “preconditioning current” was applied to polarize the electrodes, and introduce an artificial galvanic current between the cathode and anode such that the anode was protected and only corroded slightly [35, 37]. Electrochemical noise analysis, which does not involve preconditioning of electrodes and requires both identical electrodes and environments, has been proposed as an MIC monitoring approach, whereby ZRA-measured electrochemical noise is indicative of modification of metal surfaces [35, 37, 38, 58]. It has been proposed that the observation of minimal “white noise” in ZRA measurements could be indicative of uniform corrosion (i.e. our uninoculated incubations), while bursts of current (regardless of which direction) could be indicative of localized corrosion [60]. By allowing the biofilm to protect the metal in the inoculated chamber (WE1) and not preconditioning the metals, the split-chamber setup has the advantage of separating different environments, and allowing us to monitor the flow of electrons from an area protected by a biofilm to an uncovered area of metal. As such, this work may provide mechanistic understanding and a monitoring method for corrosion of metals that span redox regimes, geochemical conditions (e.g. pipes) or experience conditions of alternating O_2_ availability (e.g. coastal structures).

## Supporting Information

S1 Fig Split Cell set up of Al-Cu, where Al is WE 1 (A), and Cu is WE 2 (B). (TIFF)

S2 Fig Al-Cu. Coupled current and E_Coupl_ readings using the ZRA technique where Al is WE 1(A) and Cu is WE 2 (B). E_Coupl_ is in blue (mV_SCE_), while coupled current is in black.
(TIFF)

S3 Fig E_Coupl_ of all the environments for the ZRA experiments. (TIFF)

S4 Fig E_Coupl_ of all controls used for the ZRA experiments. (TIFF)

S1 Table Fluid Chemistry of ZRA Experiments. (DOCX)
