# Supplementary figures and images for: Use of an Electrochemical Split Cell Technique to Evaluate the Influence of Shewanella oneidensis Activities on Corrosion of Carbon Steel
Source: PLoS One. 2016 Jan 29;11(1):e0147899. doi: 10.1371/journal.pone.0147899 (PMC4733109; doi:10.1371/journal.pone.0147899)

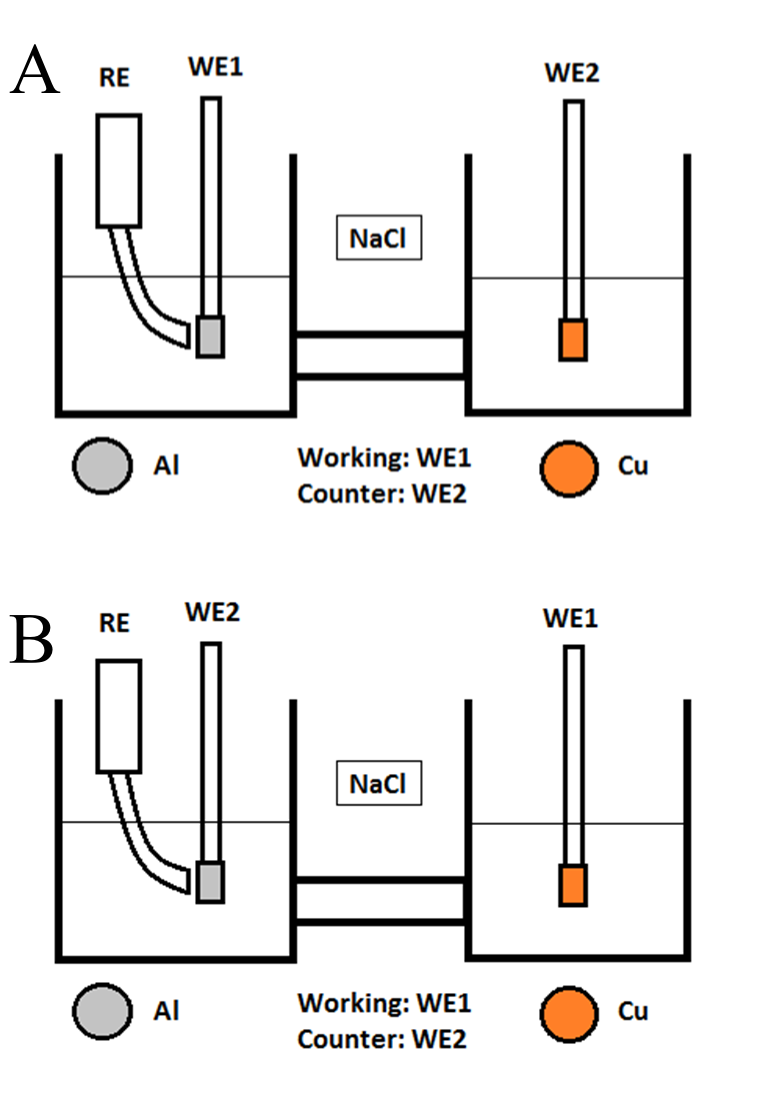

Supplement: S1 Fig — (TIFF) [file pone.0147899.s001.tiff]

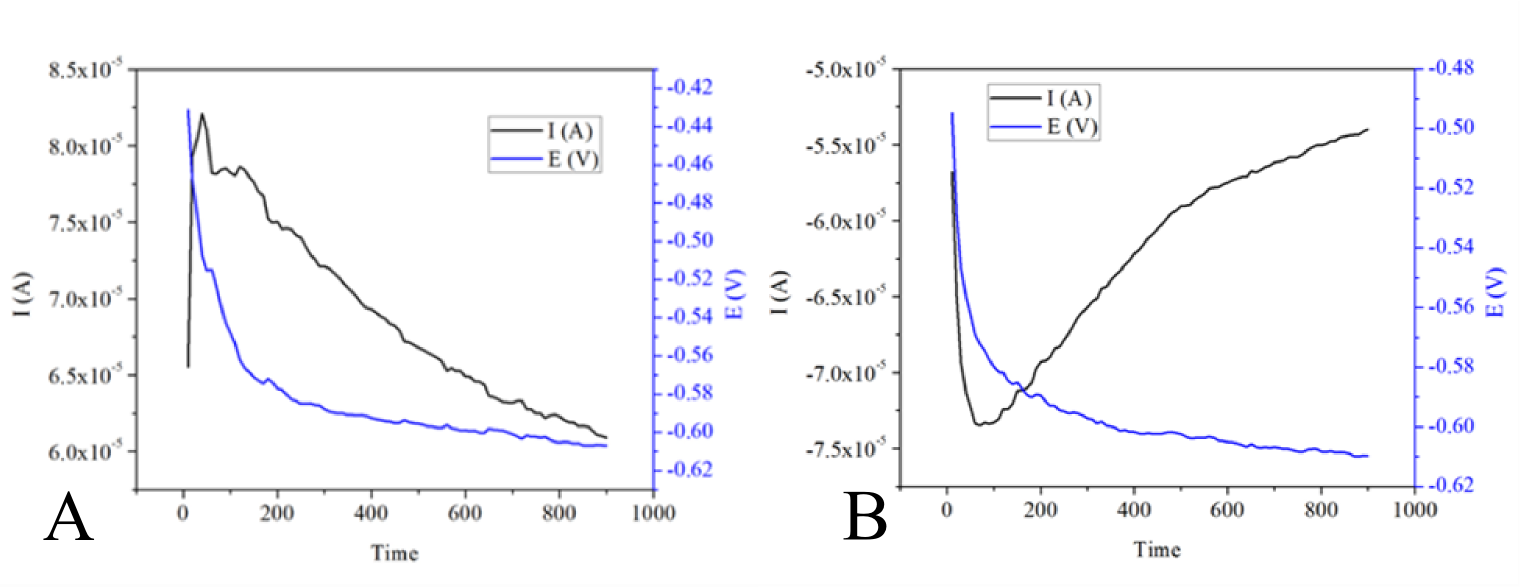

Supplement: S2 Fig — Coupled current and ECoupl readings using the ZRA technique where Al is WE 1(A) and Cu is WE 2 (B). ECoupl is in blue (mVSCE), while coupled current is in black. (TIFF) [file pone.0147899.s002.tiff]

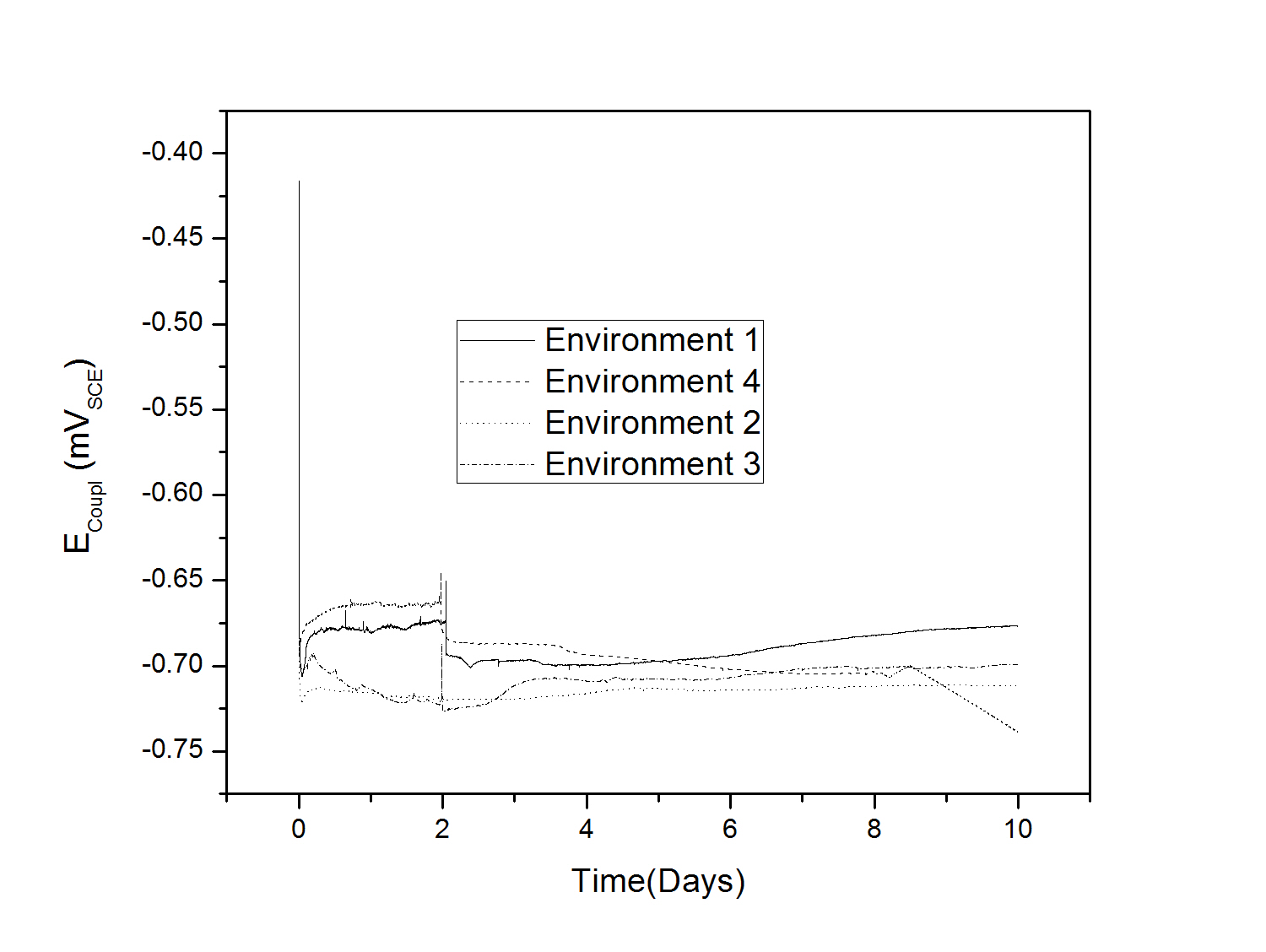

Supplement: S3 Fig — (TIFF) [file pone.0147899.s003.tiff]

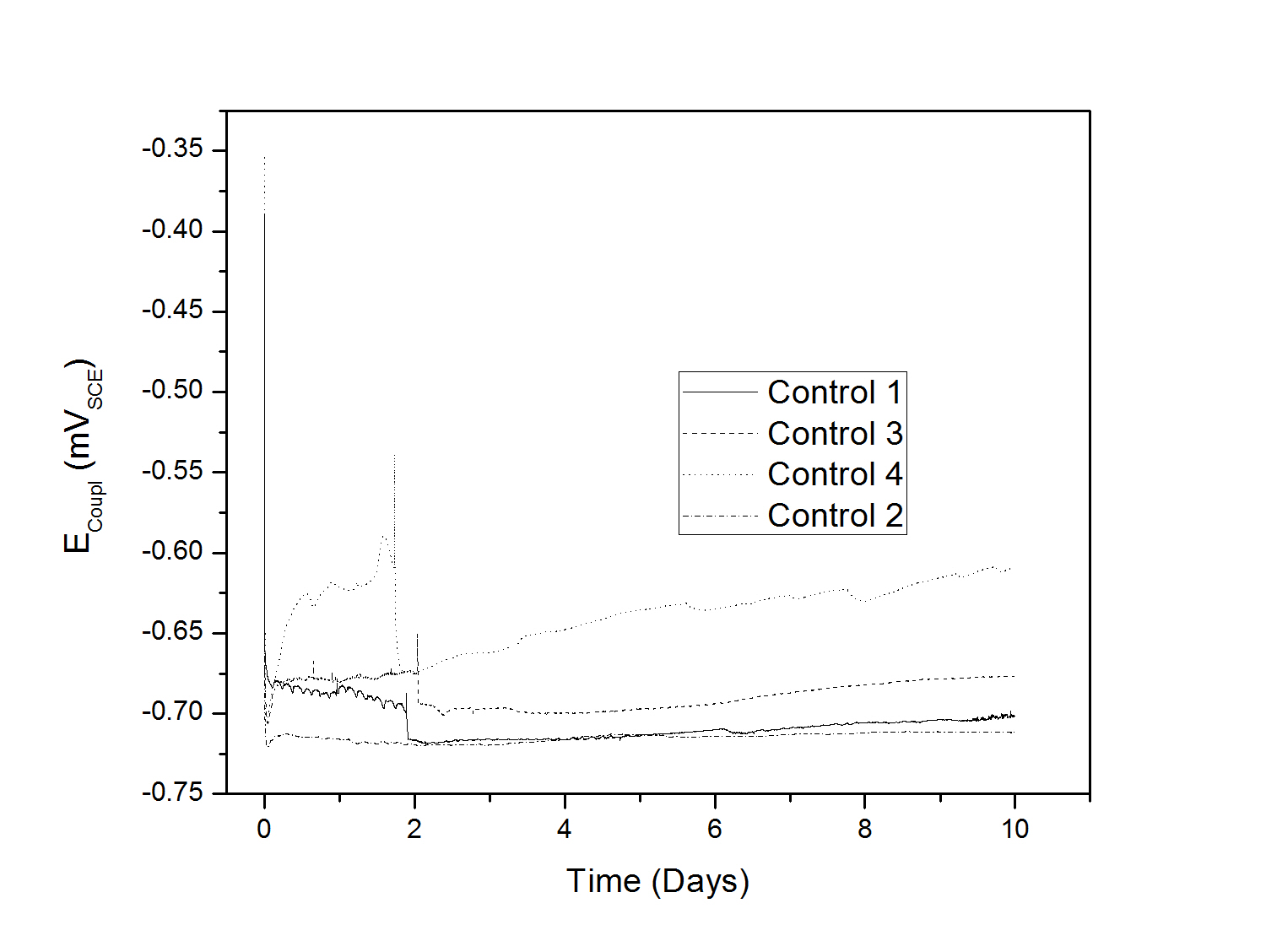

Supplement: S4 Fig — (TIFF) [file pone.0147899.s004.tiff]
