# Supplementary material for: Use of an Electrochemical Split Cell Technique to Evaluate the Influence of Shewanella oneidensis Activities on Corrosion of Carbon Steel
Source: PLoS One. 2016 Jan 29;11(1):e0147899. doi: 10.1371/journal.pone.0147899 (PMC4733109; doi:10.1371/journal.pone.0147899)
Supplement: S1 Table — (DOCX) [file pone.0147899.s005.docx]

| Environment | | Carbon Source | Inoculation | Inoculation Time (hr) | Delta pH | Max Fe^2+^( µM) | Lactate depletion (days) |
| --- | --- | --- | --- | --- | --- | --- | --- |
| 1 | WE 1 | Lactate | *S. oneidensis* | 48 | 0.18 | 54 | 3 |
|  | WE 2 | Lactate | Sterile | N/A | 0.14 | 7 | N/A |
|  | WE 1 | Lactate | *S. oneidensis* | 48 | 0.21 | 65 | 3 |
|  | WE 2 | Lactate | Sterile | N/A | 0.18 | 8 | N/A |
| 2 | WE 1 | Lactate | *S. oneidensis* | 0 | 0.17 | 51 | 3 |
|  | WE 2 | Lactate | Sterile | N/A | 0.14 | 13 | N/A |
| 3 | WE 1 | None | *S. oneidensis* | 48 | 0.17 | 43 | N/A |
|  | WE 2 | None | Sterile | N/A | 0.18 | 10 | N/A |
| 4 | WE 1 | None | *S. oneidensis* | 0 | 0.19 | 49 | N/A |
|  | WE 2 | None | Sterile | N/A | 0.08 | 12 | N/A |
| 5 | WE 1 | Lactate (additional) | *S. oneidensis* | 48 | 0.27 | 75 | 3 |
|  | WE 2 | Lactate | Sterile | N/A | 0.11 | 11 | N/A |
| Control 1 | WE 1 | Lactate | *S. oneidensis* | 48 | 0.18 | 71 | 3 |
|  | WE 2 | Lactate | *S. oneidensis* | 48 | 0.18 | 66 | 3 |
| Control 2 | WE 1 | Lactate | *S. oneidensis* | 0 | 0.3 | 62 | 3 |
|  | WE 2 | Lactate | *S. oneidensis* | 0 | 0.3 | 59 | 3 |
| Control 3 | WE 1 | Lactate | Sterile | N/A | 0.03 | 12 | N/A |
|  | WE 2 | Lactate | Sterile | N/A | 0.05 | 15 | N/A |
|  | WE 1 | Lactate | Sterile | N/A | 0.07 | 14 | N/A |
|  | WE 2 | Lactate | Sterile | N/A | 0.1 | 9 | N/A |
| Control 4 | WE 1 | None | Sterile | N/A | 0.07 | 16 | N/A |
|  | WE 2 | None | Sterile | N/A | 0.09 | 7 | N/A |
